# Prevalence and characteristics of hereditary non-polyposis colorectal cancer (HNPCC) syndrome in immigrant Asian colorectal cancer patients

**DOI:** 10.1186/s12885-017-3799-y

**Published:** 2017-12-13

**Authors:** Jasmine Lee, Yin-Yi Xiao, Yan Yu Sun, Jasminka Balderacchi, Bradley Clark, Jatin Desani, Vivek Kumar, Angela Saverimuthu, Khin Than Win, Yiwu Huang, Yiqing Xu

**Affiliations:** 1 Division of Hematology and Oncology, Department of Internal Medicine, Maimonides Medical Center, 6300 8th Avenue, Brooklyn, NY 11220 USA; 2 Department of Health Policy and Management, Graduate School of Public Health, University of Pittsburgh, Pittsburgh, PA 15261 USA; 3 Department of Pathology, Maimonides Medical Center, 4802 10th Avenue, Brooklyn, NY 11219 USA; 4 CBLPath, 760 Westchester Avenue, Rye Brook, NY 10573 USA; 5 Woman’s Health Labs, 3495 Hacks Cross Road, Memphis, TN 38125 USA; 6 Meridian Medical Group-Specialty Care, 1100 Route 72 West, Suite 201, Manahawkin, NJ 08050-2446 USA; 7 Department of Internal Medicine, Brigham and Women’s Hospital, 75 Francis Street, Boston, MA 02115 USA; 8 Private Practice, 115 St Nicholas Avenue, Brooklyn, NY 11237 USA

**Keywords:** Hnpcc, Asian, Colorectal cancer, Lynch syndrome, Screening

## Abstract

**Background:**

The prevalence of Hereditary Non-Polyposis Colorectal Cancer (HNPCC) is 2 to 5% in the Caucasian population. HNPCC is caused by genomic mutations in DNA mismatch repair genes (MMR), namely *MLH1, MSH2, MSH6, PMS2,* and *EPCAM*. A non-hereditary, acquired process of hypermethylation of the *MLH1* promoter can also lead to silencing of MLH1 protein expression. Diagnosis of HNPCC in patients with colorectal and other related cancers is important in the clinical treatment and surveillance of related cancers. The prevalence and clinical characteristics of HNPCC in Asian colorectal cancer patients has been reported in small studies and unique features have been suggested.

**Methods:**

We retrospectively reviewed the clinical characteristics of Asian patients who were diagnosed of colon cancer between 1/2002 and 6/2015, and performed IHC for four MMR protein expressions on tumor specimens as a screening test for HNPCC, followed by confirmatory tests of genomic sequencing and hypermethylation analysis.

**Results:**

One hundred forty-three patients were identified. Thirty-one patients were diagnosed younger than 50 years old, while 112 patients were diagnosed older than 50 years old. Six cases of HNPCC were found with a prevalence of 4.19%. The prevalence in the group of patients diagnosed younger than 50 years old is 16.1%, and that in patients diagnosed older than 50 years old is 0.89%. All patients with HNPCC had family histories of colon or gastric cancer. Tumor locations in the HNPCC patients were predominantly in the descending or sigmoid colon (67%). Half of the HNPCC patients had *MSH6* mutations. Hypermethylation of the *MLH1* gene was only present in 2.80% of the patients.

**Conclusion:**

The prevalence of HNPCC is high in patients younger than 50 years old and extremely low in those older than 50 years old. These results may be useful in the future development of guidelines for HNPCC laboratory screening among Asian patients. The pathological and clinical features of HNPCC in this group of Asian immigrant patients are more similar to those reported on Asian patients in their home countries than to Caucasian patients in Western countries, and will warrant further large-scale evaluation.

**Electronic supplementary material:**

The online version of this article (10.1186/s12885-017-3799-y) contains supplementary material, which is available to authorized users.

## Background

Hereditary Non-Polyposis Colorectal Cancer (HNPCC) or Lynch Syndrome is an inherited cancer susceptibility syndrome that predisposes individuals to colorectal cancer as well as extra-colonic cancers, such as endometrial, gastric, ovarian, urothelial and others, with an onset usually before 50 years old [1]. It is characterized by germ-line mutations in one of the several DNA mismatch repair (MMR) genes, namely *MLH1*, *MSH2*, *MSH6*, *PMS2*, and *EPCAM*. Mutations in *MLH1* and *MSH2* are the most common, encompassing more than 90% of all cases [2].

The mutations in MMR genes lead to defects in DNA repair. Tumors with MMR-deficiency status exhibit a high frequency of microsatellite instability (MSI-H), causing particularly unstable regions of the genome to be susceptible to errors that do not get corrected. The National Cancer Institute (NCI) recommends a microsatellite panel of five markers (NCI panel) for determining MSI status [3], and MSI-H tumors are defined as having instability in two or more markers. Tumor MMR status can also be determined by immunohistochemical (IHC) analysis of the protein products of *MLH1, MSH2, MSH6* and *PMS2*. While the gold standard for HNPCC diagnosis is genomic sequencing, screening strategies testing for loss of protein expression by IHC versus polymerase chain reaction detection of the MSI panel are considered to be equivalent [4, 5].

Hypermethylation of the promoter of the *MLH1* gene, a somatic epigenetic change, can also lead to MSI-H, and contributes to about 15% of colon cancer cases [4, 6, 7]. The *BRAF* c.1799 T > A (V600E) mutation is present in 40% of MSI-H tumors [8], and almost all are associated with hypermethylation of the *MLH1* promoter [9].

Tumors with MSI-H (somatic or germline) status have unique clinical pathological features [10]. Histologically, it can present with mucin-rich, signet ring features, and have an increased number of tumor-infiltrating lymphocytes [11]. Prognostically, patients with HNPCC have a more favorable stage-matched overall survival, but do not benefit from 5-Fluorouracil-based adjuvant chemotherapy [12]. More recently, MSI-H, associated with high mutation burden, has become a new biomarker for success of checkpoint inhibitor immunotherapy in various types of metastatic tumors [13].

Based on the Hampel et al. study, which is predominantly composed of Caucasian patients, as well as the study conducted by Aaltonen et al. in Finland, the prevalence of HNPCC is about 2.2–2.7% in colorectal cancer patients [7, 14]. In the study by Hampel et al., 23 probands were found to have HNPCC; 10 were more than fifty years of age and only 3 fulfilled the Amsterdam criteria for the syndrome [7]. Current National Comprehensive Cancer Network (NCCN) guidelines recommend screening for HNPCC for all colon cancer patients diagnosed at an age younger than 70 years old [15]. Clinical screening criteria, the Amsterdam criteria, and the Bethesda criteria [16] are not considered sensitive enough to capture all cases of HNPCC.

The clinical pathological features of HNPCC in Asian colorectal cancer patients have been studied and various features have been reported, such as early age onset, left side dominance, and the most common extra-colonic cancer being gastric cancer [17–22]. Asian American immigrants may encounter changes in diet after arriving to the United States, including variations in the quantities of meat and vegetables consumed. They may also benefit from the U.S. Preventive Services Task Force recommendation to start colonoscopy screening at fifty years old. Our hospital, Maimonides Medical Center, serves a large body of the Asian immigrant population. In this study, we evaluated the prevalence, clinical characteristics, and mutation preferences of HNPCC in immigrant Asian patients newly diagnosed of colorectal cancer.

## Methods

### Patients

This is a single-center study launched in 2009 as a retrospective analysis of patients diagnosed with colorectal cancer between 1/2002 and 12/2009. A subsequent modification was made to extend the study to include patients diagnosed between 1/2009 and 6/2015 and data was collected prospectively. The original study protocol and subsequent modifications were all approved by the Institutional Review Board (IRB) of Maimonides Medical Center (MMC). Eligibility criteria included: (1) patients of Asian origin, (2) who had a diagnoses of colon or rectal cancer, and (3) who had tumor specimens available at MMC for testing, or who had test results available from an outside clinical or reference lab. Clinical characteristics and family history were obtained from medical records. A positive family history, as defined in the revised Bethesda criteria, requires one of the patient’s first-degree relatives to be diagnosed with an HNPCC-related tumor less than 50 years of age, or two or more first- or second-degree relatives, regardless of age. The HNPCC-related cancers were defined as colorectal, endometrial, stomach, ovarian, pancreas, ureter and renal pelvis, biliary tract, brain, sebaceous gland adenomas, and small bowel cancers according to the revised Bethesda criteria [16].

### Testing for HNPCC

The screening tests for HNPCC were carried out at Maimonides Medical Center and utilized immunohistochemistry staining (IHC). The IHC was carried out in the research lab on the first 78 patients. After the revision of the NCCN guidelines to test all colon cancer patients diagnosed younger than seventy years old, the IHC test was later routinely performed in the hospital’s pathology department laboratory or outside commercial labs for 59 other patients. Three patients were tested with germline blood test first.

IHC performed in the research lab followed manufacturer’s instructions. The primary antibodies were obtained from the following commercial sources: *MLH1* [Clone ES05, Dako Inc., Carpinteria, CA), *MSH2* (Clone FE11, CalBioChem Inc., San Deigo, CA), *MSH6* (Clone 44, BD Biosciences, San Jose CA), and *PMS2* (Clone A16–4, BD Biosciences, San Jose CA). The secondary antibody was MACH 3 Mouse Probe (Biocare Medical Inc., Concord CA), and the IHC slides were developed following incubation with MACH 3 mouse HRP-polymer, and addition of DAB substrate (Dako Inc., Carpiteria, CA). Hematoxylin was then applied to the slides for counterstaining.

The tumor specimen slides were examined by the pathologist to ensure inclusion of tumor tissue. Each staining procedure included two negative controls: (1) no first antibody, and (2) a specimen of known deficiency of the particular MMR protein. The nuclear staining in the adjacent normal mucosa or the stromal tissue or lymphoid cells was used as the internal positive control.

At the setup of IHC in the research lab, a concordance study was also done by performing IHC on tumor samples known for negative or positive expressions of each of the MLH1, MSH2, MSH6 and PMS2 proteins based on germline test results, and on supplemental slides provided by the clinical laboratory at Ohio State University.

For IHC testing performed in the hospital’s pathology department laboratory, the primary antibodies used are as follows: *MLH1* (Clone ESO5, Novocastra, Buffalo Grove, IL), *MSH2* (Clone G12–1129, Cell Marque, Rocklin CA), *MSH6* (Clone 44, Cell Marque, Rocklin CA), and *PMS2* (Clone A16–4, Novocastra, Buffalo Grove, IL).

In selected patients, testing for hypermethylation of the *MLH1* gene promoter was performed at ARUP Laboratories (Salt Lake City, UT). Furthermore, all cases of HNPCC were confirmed by genomic sequencing performed at Myriad Genetics (Salt Lake City, UT) or Ambry Genetics (Aliso Viejo, CA). In selected patients with strong clinical characteristics of HNPCC, genomic testing was used as a first and definitive test per treating physicians.

## Results

### Patient characteristics

A total of 243 patients who were diagnosed of colorectal cancer between January 2002 and June 2015 were screened; 143 patients were found to be eligible and were included in the analysis. All but one patient is of Chinese descent, except for one who is of Vietnamese descent. There are 80 male and 63 female patients. The age range for diagnosis is 23 to 87 years, with a median age of 62 years (Table 1). Thirty-one patients were diagnosed at an age younger than 50 years old, and 112 patients were diagnosed older than 50 years old. Ten patients were younger than 40 years old at the time of diagnosis.

**Table 1:** Demographic characteristics of study population

Variables	Total population 143 (%)	Below 50 years *N* = 31 (%)	50 years and above *N* = 112 (%)
Confirmed HNPCC cases	6 (4.19)	5 (16.1)	1 (<1)
Gender			
Male	80 (55.9)	15 (48.4)	65 (58.0)
Female	63 (44.1)	16 (51.6)	47 (42.0)
Median age at diagnosis in years (range)	62 (23–87)	43 (23–49)	64 (50–87)

One hundred twenty-three out of the entire cohort of 143 patients had documentation showing that they were not born in the United States. One hundred twenty patients were confirmed to be immigrants from mainland China, two from Malaysia (of Chinese descent), and one from Vietnam. The data on the number of years since immigration is known for 26 patients, which ranged from four to forty years, with a median of ten years.

One hundred patients were excluded due to lack of tumor specimen for analysis, lack of clinical data, or patient refusal (*n* = 2). Their age distribution was 38–97 with a median age of 65.5 years. Among them, 10 patients are younger than 50 years of age, and 90 patients are older than 50 years of age. The tumor characteristics of the 98 ineligible patients are summarized in Additional file 1: Table S1 included in the Additional file.

### Characteristics of the HNPCC patients

Six patients were confirmed to have HNPCC, representing a prevalence of 4.19% in this cohort. Five were in the younger patient group (<50 years old), which accounts for a prevalence of 16.1% in this study. Only one patient from the older patient group was diagnosed of HNPCC, resulting in a prevalence of 0.89% for patients older than 50 years old (Table 1). This patient was diagnosed at age 71, and also happens to be the first proband in the family. Her son was diagnosed of colon cancer at 47 years old from a screening colonoscopy.

The tumor locations and stages at diagnosis are summarized in Table 2. Two-thirds of the tumors are present in the left side of the colon in the group with HNPCC, which is similar to the representation in the non-HNPCC group as well.

**Table 2:** Tumor characteristics of study population

Characteristics	HNPCC *N* = 6 (%)	Non-HNPCC *N* = 137 (%)
Tumor location		
Right side	**2 (33.3)**	**32 (23.4)**
Ascending colon	1 (16.7)	24 (17.5)
Transverse colon	1 (16.7)	7 (5.11)
Left side	**4 (66.7)**	**88 (64.2)**
Descending colon	1 (16.7)	16 (11.7)
Sigmoid colon	3 (50.0)	43 (31.4)
Rectosigmoid	0 (0)	8 (5.84)
Rectum	0 (0)	21 (15.3)
Colon, unspecified	0 (0)	12 (8.76)
Overlapping	0 (0)	6 (4.38)
Tumor stage		
Stage I	0 (0)	18 (13.1)
Stage II	5 (83.3)	39 (28.5)
Stage III	1 (16.7)	34 (24.8)
Stage IV	0 (0)	23 (16.8)
Unknown	0 (0)	23 (16.8)

The pathological and clinical characteristics of individual HNPCC patients are shown in Table 3. Five patients were diagnosed of Stage II cancer, and one was diagnosed of Stage III cancer. Tumor locations included 4 in the descending or sigmoid colon, 1 in the transverse colon and 1 in the ascending colon. At a median follow up of 27 months after diagnosis (range 9 to 85 m), two patients were lost for follow up, and one of them was diagnosed of recurrence of disease.

**Table 3:** Clinical characteristics of patients with MMR deficiency and confirmed HNPCC (*N* = 6)

Patient No.	Age	Site of Colorectal Cancer	Family History	Bethesda Criteria Met	MMR Protein Deficiency by IHC	Genomic Sequencing	Nucleotide Change	Amino Acid Change	Mechanism	Stage	Adjuvant Treatment	Survival and follow-up (as of Jan 2017)
139	28	Sigmoid	Maternal grandfather had resected colon mass at age 40	Yes #1	MSH6	MSH6	c.4002-2A>G^a^	n/a	Intervening sequence, RNA processing	T3 N0 Stage IIA	Clinical trial E5202	85 m alive
170	33	Sigmoid	Father colon cancer at age < 40 y; Maternal grandmother colon cancer at age < 40	Yes #1, #4, #5	Not done	EPCAM MLH1 MSH2	3′ terminal deletion c.1100C > A deletion exon 1	Thr367 Asn	Large deletion of 3′ EPCAM and 5′ of MSH2	T4bN0 Stage IIC	Oxaliplatin + Xeloda	65 m alive
175	43	Transverse	Father died from gastric cancer, diagnosed at 53	Yes #1	MSH2, MSH6	MSH2	c.1386 + 2 T>C^b^	n/a	Intervening sequence, RNA processing	T4aN1b Stage IIIB	Oxaliplatin 5-Fu Leucovorin	53 m (33 m since last f/u)
200	47	Sigmoid	Mother colon cancer at age 71	Yes #1	MSH6	MSH6	c.1882delT	Trp628 Glysfs^c^7	Premature truncation of the amino acid at position 634	T3 N0 Stage IIA	None	22 m alive
226	71	Ascending	Son colon cancer at age 47	Yes #4	MSH6	MSH6	c.1882delT	Trp628 Glysfs^c^7	Premature truncation of the amino acid at position 634	T3 N0 Stage IIA	None	32 m alive
275	42	Descending	Father colon cancer at age 35	Yes #1, #4	MLH1, PMS2	MLH1	c.677G > Ac	Arg226 Gln	Skipping of exon 8	T3 N0 Stage IIA	None	41 m (32 m since last f/u) Recurrence April 2014

^a^The MSH6 mutation c.4002-2A > G consists of a nucleotide substitution in a non-coding intervening sequence (IVS) occurring two nucleotides from the beginning of exon 10 [30]

^b^The MSH2 variant c.1386 + 2 T > C consists of a nucleotide substitution in a non-coding intervening sequence (IVS) occurring two base pairs from the end of exon 8 [30]

^c^The germline MLH1 mutation c.677G > A consists of a nucleotide substitution immediately adjacent to intron 8 [30]

One patient had a c.677G > A mutation on the MLH1 gene; another 2 patients had MSH2 mutations, while 3 patients had MSH6 mutations, among which 2 were from the same family (Table 3). One of the 6 patients revealed two deleterious mutations, which included a deletion in exon 1 of the 5′ region of MSH2, as well as an additional EPCAM 3′ terminal deletion.

### The sensitivity of screening with the revised Bethesda criteria on family history

In the subset of patients who had early onset of colorectal cancer (diagnosed before 50 years of age), 5 patients were confirmed to have HNPCC, and all 5 had family history of HNPCC-related cancers, mainly colon cancer. Only two patients met the family history criteria defined by the revised Bethesda guidelines, as they had first-generation relatives diagnosed with cancer at ages younger than 50 years old (Table 4). The other three patients did not meet the criteria on family history due to having only one second-degree relative with colon cancer, or having a first-degree relative diagnosed older than 50 years old, the latter of which is the case for two patients. In the older patient group, the only HNPCC patient had family history due to her son having colon cancer. None of the four patients who had MLH1 hypermethylation in the tumor had family histories of colon cancer.

**Table 4:** Analysis with the revised Bethesda criteria

	<50 *N* = 31	≥50 *N* = 112
HNPCC	*N* = 5	*N* = 1
Meeting revised Bethesda criteria #1	5	0
Meeting revised Bethesda criteria #2	0	0
Meeting revised Bethesda criteria #3	4^a^	0
Meeting revised Bethesda criteria #4	2	1
Meeting revised Bethesda criteria #5	1	0
Amsterdam criteria met	0	0
Non-HNPCC or Sporadic	*N* = 26	*N* = 111
Meeting revised Bethesda criteria #1	26	0
Meeting revised Bethesda criteria #2	0	6
Meeting revised Bethesda criteria #3	2	0
Meeting revised Bethesda criteria #4	0	0
Meeting revised Bethesda criteria #5	1	1
Amsterdam criteria met	0	0

^a^Four HNPCC patients diagnosed under age 50 show colorectal cancers with MSI-H history. The other HNPCC patient under age 50 did not have IHC or MSI testing

The revised Bethesda criteria: 1. Colorectal cancer diagnosed at less than 50 years of age. 2. Presence of synchronous, metachronous colorectal or other HNPCC associated tumors regardless of age. 3. Colorectal cancer with MSI-H history in a patient diagnosed at less than 60 years old. 4. Colorectal cancer diagnosed in one or more first degree relatives with an HNPCC related tumor with one of the cancers being diagnosed under age 50 years. 5. Colorectal cancer diagnosed in two or more first- or second-degree relatives with HNPCC-related tumors, regardless of age [16]

### Sensitivity and specificity of the revised Bethesda criteria for screening (Table 5)

If the revised Bethesda criteria are used as a screening tool, then criterion #1 pertaining to the age of onset of less than 50 years old performed best with 83% sensitivity and 81% specificity. The rest of the clinical criteria demonstrated low sensitivity but high specificity, with criterion #2 being the least sensitive. The criterion #3 using MSI testing showed 100% sensitivity. One patient had a false positive result, so the specificity is 96.6%. Using fulfillment of any of the clinical criteria, the sensitivity is 100%, which is as good as the lab test criterion.

**Table 5:** Sensitivity and specificity of the revised Bethesda criteria as a screening tool for HNPCC

Revised Bethesda criteria	Sensitivity	Specificity
#1	83.3%	81.0%
#2	0.00%	94.9%
#3	100%	96.6%
#4	50.0%	100%
#5	16.7%	98.5%
#1, or 2, or 3, or 4, or 5 (any one of the 5)	100%	75.9%

### MLH1 promoter methylation status

By IHC screening, five additional patients were found to lack expression of MMR genes, and four of them showed hypermethylation of the MLH1 gene promoter. For three of these patients, the hypermethylation test was the next test performed. For the two other patients, who had tested normal for germline mutations, hypermethylation analysis was performed as the third test. The ages for those four patients showing hypermethylation were 61, 77, 74 and 40 years old (Table 6). Their tumor locations were in the ascending colon (2 patients) and sigmoid colon (2 patients). The rate of hypermethylation of MLH1 was only 2.80% in the entire group, and 2.67% in the older patient group.

**Table 6:** Colon cancer patients with MMR deficiency detected by IHC, but confirmed to be not HNPCC (*N* = 5)

Patient No.	Age	Site of Colorectal Cancer	Family History	Bethesda Criteria Met	MMR Protein Deficiency by IHC	Genomic Sequencing	MLH1 Promoter Methylation	Stage	Adjuvant Treatment	Survival and follow-up (as of Jan 2017)
102	61	Sigmoid	None	No	PMS2	Not performed	Detected	T4N0M1 Stage IV	None	(n/a since 2007)
127	77	Ascending	None	No	MLH1, PMS2	Not performed	Detected	T3 N0 Stage IIA	None	77 m (54 m since last f/u)
224	34	Sigmoid	None	Yes #1	MLH1, MLH2	A variant of unknown significance in APC gene: c.95A > G	Not Detected	T4aN2 Stage IIIC	Oxaliplatin5-FuLeucovorin	26 m alive (1 m since last f/u) Recurrence March 2016
235	74	Ascending	None	Yes #2	MLH1, PMS2	None detected	Detected	T3N1 Stage IIIB	None	23 m alive (0 m since last f/u)
281	40	Sigmoid	None	Yes #1	MLH1, PMS2	None detected	Detected	T4aN0 Stage IIB	None	48 m alive (3 m since last f/u)

### Other genomic variants

Extensive testing was performed on the 34-year-old patient who lacked expression of MLH1 and MLH2. Results included a normal genomic test, normal hypermethylation test of MLH1, wild-type BRAF, and a variant (c.95A > G) of unknown significance in the APC gene (Table 6).

### Characteristics of the very young patients (<40 years old)

Ten patients were found to have an onset of disease before 40 years old, and the youngest patient in this group was 23 years old. Eight patients had IHC testing performed first, whereas two went directly to genomic testing (Additional file 1: Table S2). Two HNPCC cases were detected, as well as additional variants of unknown significance in the APC, MLH1, and MSH6 genes found among three other patients. One patient was found to have one copy of the MYH mutation (c.934-2A > G) which is considered deleterious. However, due to the autosomal recessive nature of the MYH-related colon cancer syndrome, this result is considered of uncertain significance.

## Discussion

In this study, we screened 243 cases of colorectal cancer in Asian immigrant patients managed in a community hospital in the South Brooklyn area of New York City, and compiled data on 143 patients with available information for HNPCC screening and confirmation. We have shown here an HNPCC prevalence of 4.19%, and a number of features that are not entirely consistent with literature. The prevalence of HNPCC in the younger population was as high as 16%, while its prevalence in patients of age 50 years and older was extremely low. The tumor locations were predominantly in the left side of the colon. The hypermethylation rate in the entire cohort and even in the older patient group was remarkably low.

To assess whether there is a difference in the clinical presentation of Asian patients with HNPCC versus Caucasian patients, we reviewed our data in the context of several published studies on Asian patients including patients from the Northern or Southern part of Mainland China [17–19, 21–23]. Strikingly, one similar result is the predominance of tumors located in the left side of the colon. Our study showed the dominant location to be in the sigmoid and descending colon (66.7%), which is a finding that was revealed in multiple other studies. Jin et al. analyzed data from hospital colorectal surgery registry and 8 patients were diagnosed of HNPCC among 158 consecutive patients. Two patients had sigmoid cancer and five had rectal cancer, while one had cancer in the hepatic flexure [17]. Liu et al. drew data from the Fudan Colorectal Registry and reported left versus right colon locations to be 64.7% versus 35.3% [23]. Furthermore, observations from the Singapore HNPCC registry reported by Chew et al. also showed that 69% of the cancers originated from the sigmoid colon [18]. On the contrary, Hampel et al. showed left side location in 43% of cases [7]. Our results are more similar to studies from Asian patients residing in Asian countries than to that from Caucasian patients in the United States. For Asian immigrants, the possible diet change or access to early screening in the U.S. may also make a difference in the clinical pathological features of HNPCC in our cohort. Although all the studies on Asian patients may suffer from the bias of small sample size, the emerging concept of left side preference of colon cancer in Asian HNPCC carriers warrants further investigation. The studies on tumor locations is especially important as recent data emerges to indicate that the location of tumors is a prognostic marker for survival, as well as a predictive marker for response to EGFR inhibitor therapy [24, 25].

Eighty-three percent of the HNPCC cases (5 out of 6) in this study were attributed to patients younger than 50 years old, and the prevalence of HNPCC was 16.1% in this age group. These results confirmed HNPCC as an early-onset, highly penetrative genetic disease. It is consistent with previous reports of relatively early onset of colorectal cancer in Asian HNPCC patients in some studies [21, 23] but not in others [18]. The average onset was 36 years old in the study by Liu et al. [23], and 37.5 years old in the Hong Kong Registry [21]. Conversely, in our study, the representation of HNPCC from the older patient group was 16.7% (1 out of 6), and the prevalence of HNPCC in the older patient group was 0.89% only. This result is different from that reported in Hampel et al., composed of a majority of Caucasian patients [7], where 43% of the total HNPCC patients were diagnosed at an age older than 50 years old. In the current revision of the NCCN guidelines, screening of HNPCC is extended to patients up to 70 years old. Additional studies will be required to further evaluate the yield of screening for HNPCC in Asian colorectal cancer patients older than 50 years old.

Ten patients (7%) were diagnosed at an age younger than 40 years old, including two diagnosed of HNPCC. Others were screened extensively for other genetic syndromes, which showed variants of unknown significance or negative findings. There is also recent publication on increasing colon cancer incidence rates in patients 20–39 years of age, from 0.5 to 1.3% annually in the United States [26]. Colorectal cancer in the very young patients is a health care issue which will warrant further epidemiology studies considering environmental factors such as diet, obesity, and lack of exercise. From this study, all of the very young Chinese cancer patients (<40 years old) were born in China and are not obese. They are presumably more likely to adopt Western diets than older patients are, but may also partially consume Eastern diets with older family members and still have relatively easy access to Eastern foods. Further studies on this subpopulation may generate useful information on the epidemiology of environmental factors associated with colorectal cancers.

Another notable finding is that all patients diagnosed of HNPCC in the study reported family history of HNPCC-related cancers. Patients of young age without family history did not have HNPCC and similarly, patients with hypermethylation of the *MLH1* promoter did not have family history. Therefore, family history is a sensitive screening tool. However, the revised Bethesda criteria would only capture three of the six HNPCC patients based on family history alone. If further relaxation is allowed on the family history criterion including age limit and the number of diagnosed relatives, then all of the patients can be recommended for screening based on family history alone. Using fulfillment of any of the revised Bethesda guidelines for screening, the sensitivity is 100% and the specificity is 76.6%, similar to that reported in other studies [27]. Among the revised Bethesda criteria, criterion #1 performed best as a single criterion with a sensitivity of 83.3%.

Mutations on *MLH1* and *MSH2* represent 90% of identifiable HNPCC cases [28]. Our study showed predominant mutations in the *MSH2* and *MSH6* genes. Similarly, Jin et al. also reported 37.5% of the mutations to be in *MSH6*, whereas *MSH6* was found in only 10% of HNPCC patients in an Australian study [20]. *MSH6* gene mutations may give rise to later age of onset and an excess of endometrial cancer, according to Wagner et al. [29]; in the family that carries *MSH6* mutations in our study, one patient developed cancer at age 71, consistent with this previous report.

Although this is a small study, our result raises the following issues to be studied in the future: (1) It will be interesting to perform epidemiological studies to analyze the potential unique interactions of gene mutations and environmental effects regarding the predominant early onset of colorectal cancers in Asian HNPCC carriers. (2) It will be highly relevant to study the cost-effectiveness of HNPCC screening in Asian patients; should laboratory testing be obtained only in those with early age of presentation and carry positive family history? In that regard, the revised Bethesda criteria performed quite well.

The limitations of this study are its retrospective nature and small sample size. We had to exclude 100 patients due to the lack of tumor specimen and clinical information or patient refusal. We were able to determine the age distribution of those patients and found a higher proportion of patients in the older patient group, which can potentially skew the results on the prevalence of colorectal cancer in the elderly.

## Conclusion

The prevalence of HNPCC in immigrant Asian colon cancer patients was 4.19%. The prevalence of HNPCC is high in patients younger than 50 years old (16%) and extremely low in those older than 50 years old. All patients with HNPCC had family history of colon or gastric cancer. The predominant tumor location is in the left side of the colon or rectum. Hypermethylation of the *MLH1* gene promoter is very rare. These results may be useful in the future development of guidelines for HNPCC laboratory screening among Asian patients. The pathological and clinical features of HNPCC in this group of immigrant Asian patients are more similar to those reported in literature about Asian patients residing in their home countries than to Caucasian patients in Western countries, and will warrant further large-scale evaluation.

## Additional file

Additional file 1: Table S1. Tumor characteristics of ineligible patients due to lack of tumor specimen for analysis. Stratified according to tumor location and stage at diagnosis. **Table S2.** Clinical characteristics of patients with early-onset of colorectal cancer, <40 years old (*N* = 10). Family history, demographic features and genetic mutations in young Asian patients (<40 years). (DOCX 24 kb)

## Abbreviations

APC: Adenomatous polyposis coli
EGFR: Epidermal growth factor receptor
EPCAM: Epithelial cell adhesion molecule
HNPCC: Hereditary non polyposis colorectal carcinoma
IHC: Immunohistochemistry staining
MLH1: MutL homolog 1
MMR: Mismatch repair
MSH2: MutS protein homolog 2
MSH6: MutS Homolog 6
MSI-H: High frequency of microsatellite instability
NCCN: National Comprehensive Cancer Network
NCI: National Cancer Institute

## References

[1] Lynch HT, de la Chapelle A (2003). Hereditary colorectal cancer. N Engl J Med.

[2] Tutlewska K, Lubinski J, Kurzawski G (2013). Germline deletions in the EPCAM gene as a cause of lynch syndrome - literature review. Hered Cancer Clin Pract..

[3] Boland CR, Thibodeau SN, Hamilton SR, Sidransky D, Eshleman JR, Burt RW, Meltzer SJ, Rodriguez-Bigas MA, Fodde R, Ranzani GN (1998). A National Cancer Institute workshop on microsatellite instability for cancer detection and familial predisposition: development of international criteria for the determination of microsatellite instability in colorectal cancer. Cancer Res.

[4] de la Chapelle A, Hampel H (2010). Clinical relevance of microsatellite instability in colorectal cancer. J Clin Oncol.

[5] Hampel H, Frankel WL, Martin E, Arnold M, Khanduja K, Kuebler P, Clendenning M, Sotamaa K, Prior T, Westman JA (2008). Feasibility of screening for lynch syndrome among patients with colorectal cancer. J Clin Oncol.

[6] Kane MF, Loda M, Gaida GM, Lipman J, Mishra R, Goldman H, Jessup JM, Kolodner R (1997). Methylation of the hMLH1 promoter correlates with lack of expression of hMLH1 in sporadic colon tumors and mismatch repair-defective human tumor cell lines. Cancer Res.

[7] Hampel H, Frankel WL, Martin E, Arnold M, Khanduja K, Kuebler P, Nakagawa H, Sotamaa K, Prior TW, Westman J (2005). Screening for the lynch syndrome (hereditary nonpolyposis colorectal cancer). N Engl J Med.

[8] Domingo E, Laiho P, Ollikainen M, Pinto M, Wang L, French AJ, Westra J, Frebourg T, Espin E, Armengol M (2004). BRAF screening as a low-cost effective strategy for simplifying HNPCC genetic testing. J Med Genet.

[9] Noushmehr H, Weisenberger DJ, Diefes K, Phillips HS, Pujara K, Berman BP, Pan F, Pelloski CE, Sulman EP, Bhat KP (2010). Identification of a CpG island methylator phenotype that defines a distinct subgroup of glioma. Cancer Cell.

[10] Gatalica Z, Vranic S, Xiu J, Swensen J, Reddy S (2016). High microsatellite instability (MSI-H) colorectal carcinoma: a brief review of predictive biomarkers in the era of personalized medicine. Familial Cancer.

[11] Smyrk TC, Watson P, Kaul K, Lynch HT (2001). Tumor-infiltrating lymphocytes are a marker for microsatellite instability in colorectal carcinoma. Cancer.

[12] Ribic CM, Sargent DJ, Moore MJ, Thibodeau SN, French AJ, Goldberg RM, Hamilton SR, Laurent-Puig P, Gryfe R, Shepherd LE (2003). Tumor microsatellite-instability status as a predictor of benefit from fluorouracil-based adjuvant chemotherapy for colon cancer. N Engl J Med.

[13] Le DT, Uram JN, Wang H, Bartlett BR, Kemberling H, Eyring AD, Skora AD, Luber BS, Azad NS, Laheru D (2015). PD-1 blockade in tumors with mismatch-repair deficiency. N Engl J Med.

[14] Aaltonen LA, Salovaara R, Kristo P, Canzian F, Hemminki A, Peltomaki P, Chadwick RB, Kaariainen H, Eskelinen M, Jarvinen H (1998). Incidence of hereditary nonpolyposis colorectal cancer and the feasibility of molecular screening for the disease. N Engl J Med.

[15] Colon Cancer. In: Clinical Practice Guidelines in Oncology (NCCN Guidelines). National Comprehensive Cancer Network. 2017. https://www.nccn.org/professionals/physician_gls/pdf/colon.pdf. Accessed 29 Nov 2017.

[16] Umar A, Boland CR, Terdiman JP, Syngal S, de la Chapelle A, Ruschoff J, Fishel R, Lindor NM, Burgart LJ, Hamelin R (2004). Revised Bethesda guidelines for hereditary nonpolyposis colorectal cancer (lynch syndrome) and microsatellite instability. J Natl Cancer Inst.

[17] Jin HY, Liu X, Li VK, Ding Y, Yang B, Geng J, Lai R, Ding S, Ni M, Zhao R (2008). Detection of mismatch repair gene germline mutation carrier among Chinese population with colorectal cancer. BMC Cancer.

[18] Chew MH, Koh PK, Ng KH, Lim JF, Ho KS, Ooi BS, Tang CL, Eu KW (2008). Phenotypic characteristics of hereditary non-polyposis colorectal cancer by the Amsterdam criteria: an Asian perspective. ANZ J Surg.

[19] Chew MH, Tan WS, Liu Y, Cheah PY, Loi CT, Tang CL (2015). Genomics of hereditary colorectal cancer: lessons learnt from 25 years of the Singapore polyposis registry. Ann Acad Med Singap.

[20] Talseth-Palmer BA, McPhillips M, Groombridge C, Spigelman A, Scott RJ (2010). MSH6 and PMS2 mutation positive Australian lynch syndrome families: novel mutations, cancer risk and age of diagnosis of colorectal cancer. Hered Cancer Clin Pract..

[21] Ho JW, Wei R, Chan EM (2005). Hereditary colorectal cancer syndromes in Hong Kong: a Registry's perspective. Hered Cancer Clin Pract.

[22] Yan HL, Hao LQ, Jin HY, Xing QH, Xue G, Mei Q, He J, He L, Sun SH (2008). Clinical features and mismatch repair genes analyses of Chinese suspected hereditary non-polyposis colorectal cancer: a cost-effective screening strategy proposal. Cancer Sci.

[23] Liu F, Yang L, Zhou X, Sheng W, Cai S, Liu L, Nan P, Xu Y (2014). Clinicopathological and genetic features of Chinese hereditary nonpolyposis colorectal cancer (HNPCC). Med Oncol.

[24] ND VAP, Innocenti F, Fruth B, Greene C, O’Neil BH, Shaw JE, Atkins JN, Horvath LE, Polite BN (2016). Impact of primary (1°) tumor location on overall survival (OS) and progression-free survival (PFS) in patients (pts) with metastatic colorectal cancer (mCRC): analysis of CALGB/SWOG 80405 (alliance). J Clin Oncol.

[25] Tejpar S, Stintzing S, Ciardiello F, Tabernero J, Van Cutsem E, Beier F, Esser R, Lenz HJ, Heinemann V. Prognostic and predictive relevance of primary tumor location in patients with RAS wild-type metastatic colorectal cancer: retrospective analyses of the CRYSTAL and FIRE-3 trials. JAMA Oncol. 2016;3:194–201. 10.1001/jamaoncol.2016.3797 PMC7505121 27722750

[26] Siegel RL, Fedewa SA, Anderson WF, Miller KD, Ma J, Rosenberg PS, Jemal A. Colorectal cancer incidence patterns in the United States, 1974–2013. J Natl Cancer Inst. 2017;109(8):djw322. 10.1093/jnci/djw322 PMC6059239 28376186

[27] Moreira L, Balaguer F, Lindor N, de la Chapelle A, Hampel H, Aaltonen LA, Hopper JL, Le Marchand L, Gallinger S, Newcomb PA (2012). Identification of lynch syndrome among patients with colorectal cancer. JAMA.

[28] Peltomaki P (2001). Deficient DNA mismatch repair: a common etiologic factor for colon cancer. Hum Mol Genet.

[29] Wagner A, Hendriks Y, Meijers-Heijboer EJ, de Leeuw WJ, Morreau H, Hofstra R, Tops C, Bik E, Brocker-Vriends AH, van Der Meer C (2001). Atypical HNPCC owing to MSH6 germline mutations: analysis of a large Dutch pedigree. J Med Genet.

[30] Shapiro MB, Senapathy P (1987). RNA splice junctions of different classes of eukaryotes: sequence statistics and functional implications in gene expression. Nucleic Acids Res.

