# Supplementary material for: Prevalence and characteristics of hereditary non-polyposis colorectal cancer (HNPCC) syndrome in immigrant Asian colorectal cancer patients
Source: BMC Cancer. 2017 Dec 13;17:843. doi: 10.1186/s12885-017-3799-y (PMC5729240; doi:10.1186/s12885-017-3799-y)
Supplement: Additional file 1: Table S1. — Tumor characteristics of ineligible patients due to lack of tumor specimen for analysis. Stratified according to tumor location and stage at diagnosis. Table S2. Clinical characteristics of patients with early-onset of colorectal cancer, <40 years old (N = 10). Family history, demographic features and genetic mutations in young Asian patients (<40 years). (DOCX 24 kb) [file 12885_2017_3799_MOESM1_ESM.docx]

**Table S1. Tumor characteristics of ineligible patients due to lack of tumor specimen for analysis (Supplemental)**

| **Characteristics** | **N = 98 (%)** |
| --- | --- |
| **Tumor location**    **Right side**  Ascending colon  Transverse colon  **Left side**  Descending colon  Sigmoid colon  Rectosigmoid  Rectum  **Colon, unspecified**    **Overlapping** | **19 (19.4)**  16 (16.3)  3 (3.06)  **65 (66.3)**  11 (11.2)  22 (22.4)  7 (7.14)  25 (25.5)  14 (14.3)  0 (0) |
| **Tumor stage**  Stage I  Stage II  Stage III  Stage IV  Unknown | 12 (12.2)  17 (17.3)  33 (33.7)  20 (20.4)  16 (16.3) |

| **Patient No.**  **Table S2. Clinical characteristics of patients with early-onset of colorectal cancer, <40 years old (n=10)** | **Age** | **Site of Colorectal Cancer** | **Family History** | **Bethesda Criteria Met** | **MMR Protein Deficiency by IHC** | **Genomic Sequencing** | **Nucleotide Change** | **Amino Acid Change** | **Clinical Significance** |
| --- | --- | --- | --- | --- | --- | --- | --- | --- | --- |
| 19 | 27 | Descending | None | Yes  #1 | None detected | Not done | n/a | n/a | n/a |
| 79 | 34 | Rectum | None | Yes  #1 | Not done | No mutation detected | n/a | n/a | n/a |
| 111 | 37 | Transverse | None | Yes  #1 | None detected | No mutation detected | n/a | n/a | n/a |
| 139 | 28 | Sigmoid | Maternal grandfather had resected colon mass at age 40 | Yes  #1 | MSH6 | MSH6 | c.4002-2A>G | n/a | HNPCC |
| 162 | 23 | Rectum | None | Yes  #1 | None detected | No mutation detected | n/a | n/a | n/a |
| 170 | 33 | Sigmoid | Father and maternal grandmother colon cancer, both at <40 years old | Yes  #1, #4, #5 | Not done | EPCAM  MLH1  MSH2 | 3’ terminal deletion  c.1100C>A  deletion exon 1 | Thr367Asn | HNPCC  Uncertain Significance  HNPCC |
| 183 | 37 | Sigmoid | Grandfather died of gastric cancer | Yes  #1 | None detected | No mutation detected | n/a | n/a | n/a |
| 224 | 34 | Sigmoid | None | Yes  #1 | MLH1  MLH2 | APC | c.95A>G | n/a | Unknown Significance |
| 238 | 34 | Transverse | None | Yes  #1 | None detected | MSH6  MYH | c.-11C>T^*^  c.934-2A>G | n/a | Uncertain Significance  Clinical Significance Undetermined |
| 306 | 36 | Rectosigmoid | None | Yes  #1 | None detected | No mutation detected | n/a | n/a | n/a |

*”The MSH6 variant c.-11C>T consists of a nucleotide substitution in the 5’ untranslated region occurring 11 base pairs upstream of the initiation codon of the MSH6 protein (Myriad Genetics).
